# Dengue and hepatitis E virus infection in pregnant women in Eastern Sudan, a challenge for diagnosis in an endemic area

**DOI:** 10.11604/pamj.2014.19.391.5439

**Published:** 2014-12-18

**Authors:** Adel Hussein Elduma, Waleed Mohammed Osman

**Affiliations:** 1 National Public Health laboratory, Ministry of Health, Sudan; 2 Department of Epidemiology, Red Sea state Ministry of Health, Sudan

**Keywords:** Dengue, HEV, pregnant women, Port Sudan

## Abstract

Dengue fever and hepatitis E virus infection are both a public health problem in developing countries due to poor sanitation. Infection with viral hepatitis and dengue fever can present with similar clinical such and fever, headache and abortion. This study was conducted in Port-Sudan city in the eastern part of the country. ELISA and Real Time PCR tests were used to detect the infection. A total number of 39 pregnant women with a mean age 26 ±7.8 were included in the study. All of them had fever, 32 (92.3%) admitted with headache, 11 (28.2%) of them had vomiting, and abortion was reported in two cases (5.1%). The study showed that 4 (10.3%) of pregnant women were positive for the Hepatitis E virus, 5 (12.8%) positive for Dengue virus IgG, and only one sample (2.6%) was positive for IgM capture ELISA and real time PCR. Death due to hepatitis E infection was reported in one case with 7^th^ month of pregnancy. Most of hepatitis cases were reported in the central sector of the Portsudan city. The diagnosis of hepatitis E virus and dengue virus in an endemic area is a great challenge for health care staff working in these areas. Both Dengue virus and Hepatitis E virus infection should be considered in pregnant women especially in similar settings.

## Introduction

Dengue fever and hepatitis E virus infection are both a public health problem in developing countries due to poor sanitation. Infection with viral hepatitis and dengue fever can present with similar clinical such and fever, headache and abortion. Dengue haemorrhagic fever and dengue fever are an arboviral disease caused by dengue virus, belong to the genus Flavivirus, family Flaviviridae. Dengue virus is a vector borne disease transmitted mainly by Aedes aegypti [1]. Dengue outbreaks have been reported in the Eastern Mediterranean Region as early as 1799 in Egypt [2]. In Africa, data regarding dengue virus outbreaks are poor, but there are many evidences which indicated that the dengue viruses 1&2 appear to be common causes of acute fever such as in Comoros and Mozambique [3]. In recent years, dengue fever and dengue haemorrhagic fever become a public health problem in the eastern part of the country and mainly portsudan city. But this disease has a long history in this area and first outbreak was reported by Balfour and Archibald in 1908 [4]. In study conducted by Hyams and. al. 1986 involved febrile cases in Portsudan, he reported a dengue virus infection in 20% of the samples collected during his study [5]. The first case of hepatitis E virus was reported in Sudan in 1992, where acute sporadic cases were found among Sudanese children. Acute infection hepatitis E (positive for IgM anti-HEV) was found in (59%) of the study population [6]. In Darfur, Sudan, acute hepatitis E virus infection was confirmed in 95% of the suspected cases with hepatitis in 2004 [7].

## Methods

The total number of 39 women admitted the Port Sudan Maternity hospital with fever symptoms were included in this study. The study duration was between January and April 2012. A.Data was analysed by using SPSS. Informed consent was taken from each participant. For laboratory diagnosis; Enzyme linked immune assay capture, ELISA, was used to detect the Dengue virus IgM and IgG from patient's serum. Commercial kit (Dengue IgM and IgG capture ELISA) was used to detect both IgM and IgG antibodies. Reaction result was read by the ELISA reader at 450 nm with reference filter of 600-650nm absorption [8]. ELISA kit (MP diagnostics) was also used to detect HEV IgM antibodies from pregnant women [9]. RNA was extracted from serum and kept at -80 C° until the PCR done. RNA was extracted by using a QIAamp viral RNA kit (QIAamp, GmHb, Germany). The result of both dengue and hepatitis E viruses were confirmed by using Real time PCR (Rotorgene 6000).

## Results

A total number of 39 pregnant women with a mean age 26 ±7.8 were included in the study. All of them had fever, 32 (92.3%) admitted with headache, 11 (28.2%) of them had vomiting, and abortion was reported in two cases (5.1%). The study showed that 4 (10.3%) of pregnant women were positive for the Hepatitis E virus, 5 (12.8%) positive for Dengue virus IgG, and only one (2.6%) were positive for Dengue IgM capture ELISA (Table 1). Real Time PCR was done for all hepatitis E and dengue (including IgM and IgG) positive samples. Death due to hepatitis E infection was reported in one case with 7th month of pregnancy. Most of hepatitis cases among pregnant women were reported in the central sector of the Portsudan city and it was statistically significant (p= 0.013) (Table 2). Two samples of hepatitis E virus were positive by Real Time PCR.

**Table 1:** Dengue and hepatitis E virus infection among pregnant women

Type of Test	Number of pregnant women	
	Positive	Negative
Dengue IgM	1 (2.5%)	38
Dengue IgG	5 (12.8%)	34
HEV	4 (10.2%)	35

**Table 2:** Distribution of positive cases in different areas of Port Sudan city

Sector	Number of pregnant women						Total
	HEV		Dengue IgM		Dengue IgG		
	Positive	negative	Positive	negative	Positive	negative	
Southern	0	18	0	18	4	14	18
Central	1	10	0	10	1	10	11
Eastern	3	7	1	9	0	10	10
Sub-total	4	35	1	37	5	34	
Total	39		39		39		39

## Discussion

Dengue virus infection and hepatitis R virus infection symptoms and clinical signs are similar and it is difficult to distinguish especially during the acute phase of infection. These medical signs and symptoms include Fever, headache, and Jaundice. A study conducted in India among pregnant woman, dengue virus infection was presented mainly with myalgia plus a few cases with fever and abdominal pain [10]. Furthermore, fever and abdominal pain were observed in pregnant women infected with hepatitis E virus in Ghana [11]. There are many risk factors enhance the viral replication and expression in women such as malnutrition, and folate deficiency [12]. In a study conducted in Pakistan found that women was infected with Dengue virus and hepatitis E virus which support our study in the ability for mixed infection [13]. Moreover, another study was conducted in India found that a young man was infected with, dengue, HEV, and Leptospira in the same time. This case was confusing to treating physician because of several overlapping of clinical features of these diseases [14]. This result was supported by another study conducted among journalist and relief workers in Somalia, 1992-1993. Dengue virus and hepatitis E infection were reported among suspected cases admitted to hospital with clinical symptoms include fever and hepatitis [15].

## Conclusion

The diagnosis of hepatitis E virus and dengue virus in an endemic area is a great challenge for health care staff working in these areas. Both Dengue virus and Hepatitis E virus infection should be considered in pregnant women especially in areas where these diseases are circulated.
